# Economic Analyses of COVID-19 Interventions: A Narrative Review of Global Evidence

**DOI:** 10.3390/healthcare13243249

**Published:** 2025-12-11

**Authors:** Ralitsa Raycheva, Kostadin Kostadinov, Vanya Rangelova, Ani Kevorkyan

**Affiliations:** 1 Department of Social Medicine and Public Health, Faculty of Public Health, Medical University Plovdiv, 4002 Plovdiv, Bulgaria; kostadinr.kostadinov@mu-plovdiv.bg; 2 Department of Epidemiology and Disaster Medicine, Faculty of Public Health, Medical University Plovdiv, 4002 Plovdiv, Bulgaria; vanya.rangelova@mu-plovdiv.bg (V.R.); ani.kevorkyan@mu-plovdiv.bg (A.K.)

**Keywords:** COVID-19, cost-effectiveness, economic evaluation, health economics, health technology assessment (HTA), pandemic preparedness, public health interventions, vaccination, testing strategies, equity

## Abstract

**Highlights:**

**What are the main findings?**

- Vaccination—especially with mRNA vaccines—is consistently the most cost-effective COVID-19 intervention, often cost-saving in high-risk populations.
- Combined strategies integrating vaccination, testing, and targeted social distancing yield superior health and economic outcomes compared with single-measure approaches.

**What are the implications of the main findings?**

- Early, targeted, and layered implementation should guide future pandemic response and preparedness strategies to maximize economic and societal value.
- Incorporating equity, indirect effects (e.g., productivity, education), and standardized methodological frameworks will strengthen decision-making and resource prioritization in future public health emergencies.

**Abstract:**

**Background/Objectives:** The coronavirus disease 2019 (COVID-19) pandemic imposed an unprecedented global health and economic burden, prompting rapid implementation of diverse public health interventions. This review aimed to synthesize global evidence on the cost-effectiveness of key COVID-19 control strategies, including vaccination, testing, and social distancing and to identify methodological, contextual, and equity-related determinants of their economic value. **Methods:** A narrative literature review was conducted using peer-reviewed studies published between January 2020 and September 2025 and indexed in PubMed, Scopus, and Web of Science. Eligible studies included economic evaluations and modeling analyses addressing COVID-19 interventions in healthcare, community, or educational settings. Data on costs, outcomes, and methodological features were extracted and synthesized descriptively. **Results:** Across 74 included studies, vaccination—particularly with messenger RNA (mRNA) platforms—emerged as the most cost-effective intervention across all settings, often cost-saving among high-risk populations. Combined or layered strategies integrating vaccination, testing, and selective social distancing consistently outperformed single interventions in both health and economic outcomes. Early and targeted implementation yielded the highest cost-effectiveness by preventing exponential transmission and healthcare overload. However, heterogeneity in modeling assumptions, analytic perspectives, and outcome measures limited comparability. Few studies applied extended or distributional cost-effectiveness frameworks to address equity, while indirect and long-term effects such as productivity losses and “long COVID” were frequently omitted. **Conclusions:** COVID-19 interventions are most efficient when early, targeted, and adaptive to local epidemiologic conditions. Integrating equity, methodological consistency, and broader societal impacts into future evaluations will strengthen evidence-based, economically sustainable pandemic preparedness and response strategies.

## 1. Introduction

Economic analyses conducted for coronavirus disease 2019 have primarily focused on cost-of-illness studies, cost-effectiveness analyses, and cost-utility analyses. These analyses have evaluated both the direct medical costs (such as hospitalization, ICU care, medications, and diagnostics) and indirect costs (including productivity losses due to morbidity, mortality, and public health interventions like lockdowns and movement restrictions) [1,2,3,4,5]. Cost-of-illness studies quantify the total economic burden of COVID-19 at national, regional, and hospital levels. In China, productivity losses due to movement restrictions accounted for the vast majority of societal costs, far exceeding direct healthcare expenditures [2]. Hospital-based analyses have shown that intensive care is a major driver of direct medical costs, with substantial additional costs from postponed elective procedures and infection control measures [4]. Cost-effectiveness analyses have assessed interventions such as social distancing, quarantine, personal protective equipment, and screening. These studies generally find that screening and social distancing are cost-effective, especially over longer time horizons, but results are highly context-dependent and sensitive to epidemiological parameters like the reproduction number [6,7]. The inclusion of non-health impacts and distributional effects remains limited, though their consideration is essential for comprehensive policy evaluation [5,8]. Economic modeling has also examined the interplay between disease transmission, mortality, and economic output, highlighting complex trade-offs between public health measures and economic activity [9,10,11].

This review summarizes existing evidence on the economic evaluation of COVID-19 interventions, comparing findings across healthcare settings and population groups. The aim is to analyze and interpret the scope, methods, and key findings of economic studies on COVID-19 focuses on vaccination, testing, social distancing, and combined strategies.

### State of the Art

Since 2020, a rapidly expanding body of economic evidence has assessed the value of COVID-19 prevention and mitigation strategies. Most studies have examined direct medical costs and short-term outcomes, with fewer addressing indirect or distributional impacts such as productivity loss, mental health, and educational disruption. Previous reviews have typically focused on single interventions—most often vaccination—or specific regions, providing limited comparative insights across testing, non-pharmaceutical interventions, and combined strategies. Moreover, methodological heterogeneity in model design, perspective, and time horizon hampers comparability and policy translation.

This review advances the field by providing an integrated synthesis of economic evaluations published between January 2020 and September 2025, encompassing healthcare, community, and educational settings. It highlights methodological trends, equity considerations, and key cost drivers shaping cost-effectiveness across intervention types. The analysis positions current economic evidence within a broader, policy-relevant framework for pandemic preparedness and health system resilience.

## 2. Materials and Methods

This narrative literature review synthesizes findings from peer-reviewed economic evaluations and epidemiological modeling studies published between January 2020 and September 2025, focusing on the economic and equity impacts of COVID-19 prevention and mitigation strategies. The review followed the methodological guidance for narrative evidence syntheses outlined in the JBI Manual for Evidence Synthesis (2024) [12]. The decision about the narrative design was based on three considerations. First, economic evaluations of COVID-19 interventions differ markedly in design, perspective, time horizon, and analytic framework, limiting the feasibility of quantitative pooling or formal meta-analysis. Second, the purpose of this work was not only to summarize numerical cost-effectiveness outcomes but also to analyze methodological patterns, data sources, and reporting practices across diverse study types. Third, the rapidly evolving and multidisciplinary nature of the literature—spanning vaccination, testing, and non-pharmaceutical interventions—necessitated an integrative, state-of-the-art synthesis that could capture emerging trends and contextual nuances beyond the scope of narrowly focused systematic reviews. This approach allows a more comprehensive understanding of the methodological maturity and policy relevance of current economic evidence.

### 2.1. Search Strategy

A comprehensive search was conducted in three electronic databases—PubMed (MEDLINE), Scopus (Elsevier), and Web of Science (Core Collection)—to identify relevant publications. Searches covered the period 1 January 2020 to 30 September 2025 and were last updated on 30 September 2025. The Boolean logic combined three key concept blocks: (1) COVID-19; (2) economic evaluation; and (3) public-health interventions (vaccination, testing, and non-pharmaceutical measures). Search syntax was tailored to each database; the complete search strings, filters, and record yields are presented in Appendix A—Database Search Strategies (Table A1).

Reference lists of included studies and relevant reviews were manually screened to ensure completeness. All citations were imported into EndNote X9 for reference management and removal of duplicate entries.

### 2.2. Inclusion and Exclusion Criteria

Studies were included if they met the following criteria:

Design: Peer-reviewed economic evaluations (cost-effectiveness, cost-utility, cost–benefit, or budget-impact analyses) or model-based studies (decision-analytic, compartmental, or microsimulation models).

- Setting: Conducted in healthcare, community, or educational environments (e.g., hospitals, long-term care facilities, schools).
- Interventions: Addressed one or more of the following—vaccination (including booster programs); testing and screening strategies (PCR, antigen, or hybrid approaches); non-pharmaceutical interventions (NPIs) such as social distancing and school closures; or combined multicomponent strategies.
- Perspective: Economic analyses conducted from a healthcare payer/provider or societal perspective.
- Outcomes: Reported incremental cost-effectiveness ratios (ICERs), net monetary benefit (NMB), or other quantifiable cost-effectiveness metrics (e.g., cases, hospitalizations, or deaths averted).
- Publication characteristics: English-language, peer-reviewed journal articles published between January 2020 and September 2025.

Studies were excluded if they (1) did not report economic outcomes (e.g., purely epidemiological or clinical studies without cost data); (2) were commentaries, editorials, policy briefs, or non-peer-reviewed sources; (3) focused solely on macroeconomic impacts without a defined health intervention; (4) lacked quantitative cost-effectiveness or budget-impact results; or (5) were duplicates or unavailable in full text.

### 2.3. Screening and Data Extraction

Two reviewers (R.R. and K.K.) independently screened all titles and abstracts, followed by full-text assessment of eligible articles. Discrepancies were resolved by consensus with a third reviewer (A.K.). Extracted data included, setting, type of intervention, methodological framework, analytic perspective, time horizon, and key economic outcomes (ICER, NMB, QALYs gained). Additional variables captured included equity considerations, non-health impacts (productivity loss, educational disruption, mental health), and compliance with the Consolidated Health Economic Evaluation Reporting Standards (CHEERS 2022) reporting standards [13].

### 2.4. Appraisal of Included Studies

To enhance transparency and interpretive validity, each included study was appraised for methodological quality using key domains derived from the Consolidated Health Economic Evaluation Reporting Standards (CHEERS 2022) and the Philips checklist for model-based economic evaluations [13,14].

The appraisal covered the following aspects:

1. Study perspective and comparators—whether the chosen perspective (healthcare, societal) and comparator were clearly justified.
2. Analytical framework—appropriateness of model type or trial design and transparency of assumptions.
3. Time horizon and discounting—consistency with the intervention’s expected duration of benefit.
4. Cost and outcome data sources—transparency and local relevance.
5. Handling of uncertainty—inclusion of deterministic or probabilistic sensitivity analyses.
6. Equity and distributional analysis—whether subgroup or DCEA/ECEA frameworks were applied.
7. Validation and calibration (for models)—evidence of internal or external validation.
8. Reporting quality—explicit alignment with CHEERS 2022 items.

Two reviewers (R.R. and K.K.) independently rated each study as adequate, partially adequate, or not reported for each domain. Discrepancies were resolved by consensus. Aggregate findings and common methodological gaps are summarized in Table 1.

## 3. Results

The database search identified 6521 records in total. After removal of 1875 duplicates, 4646 unique records were screened by title and abstract. Following screening, 178 full-text articles were assessed for eligibility, and 104 studies were excluded. A total of 74 studies met all inclusion criteria and were included in the final qualitative synthesis.

**Figure 1:**
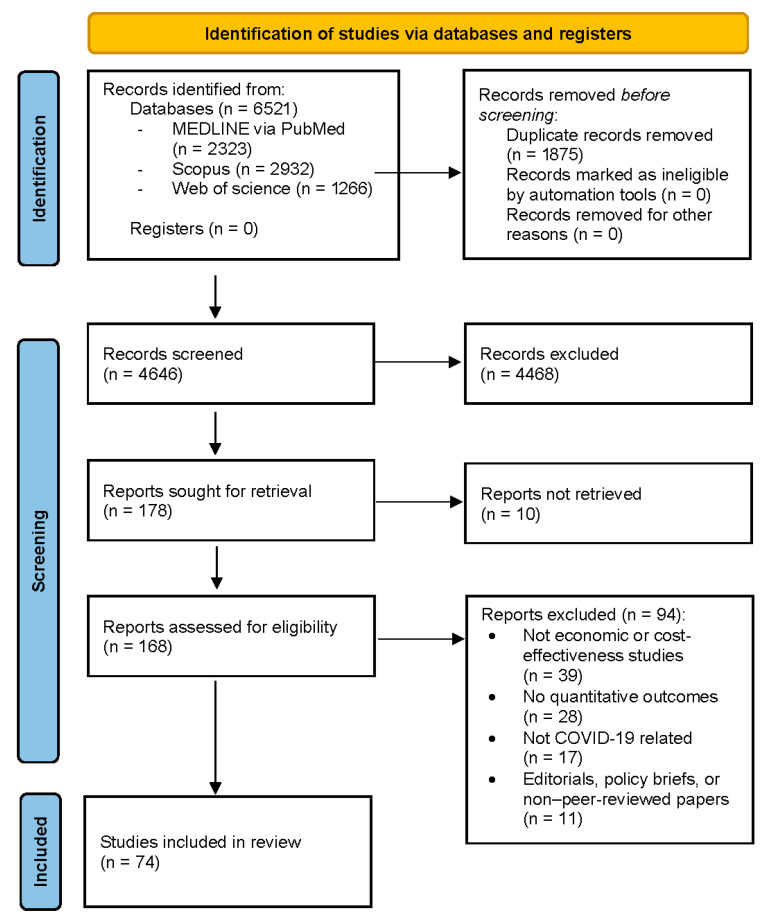
Prisma diagram of the screening process.

The articles search flow was illustrated on the PRISMA diagram (Figure 1) for the three phases of the process: identification, screening, and inclusion [15].

Table 2 summarizes the methodological characteristics of the 74 economic evaluations included in this review. These characteristics highlighted the predominance of model-based evaluations from high-income settings, substantial heterogeneity in analytic perspective and outcome measures, and limited integration of equity and long-term societal considerations. Along with these findings the methodological appraisal, based on Table 1 criteria revealed considerable variability in design quality and reporting standards across published economic evaluations of COVID-19 interventions. While most analyses clearly articulated their target population and intervention type, several methodological domains demonstrated recurrent weaknesses. More than one-third of studies lacked transparent reporting of model structure or validation procedures, and almost 30% applied short time horizons without explicit justification—potentially underestimating long-term health and economic consequences. In nearly half of the evaluations, cost and utility data were derived from secondary or non-country-specific sources, limiting contextual applicability. Probabilistic sensitivity analyses were frequently omitted, constraining the assessment of parameter uncertainty. Equity considerations were particularly underrepresented, with only about one-fifth of studies addressing differential impacts across population subgroups or employing distributional cost-effectiveness frameworks.

Across studies, vaccination strategies were consistently cost-effective in both high- and middle-income settings, with incremental cost-effectiveness ratios (ICERs) typically below country-specific willingness-to-pay thresholds. Testing and screening interventions showed more variable results, depending on test frequency, prevalence, and the presence of confirmatory PCR. Non-pharmaceutical interventions (e.g., social distancing, mask mandates) demonstrated cost-saving effects primarily when implemented early or in combination with vaccination. Integrated approaches that combined vaccination, testing, and behavioral measures yielded the most favorable outcomes in probabilistic models.

These structured findings form the basis for the interpretive synthesis that follows, which discusses methodological quality and contextual variation across settings. The Advisory Committee on Immunization Practices (ACIP) in the United States reports incremental cost-effectiveness ratios (ICERs) of $23,308 per QALY for adults ≥ 65 years, with higher ICERs in younger age groups but still within accepted thresholds when accounting for higher risk or lower vaccine cost [16]. Systematic reviews confirm that mass vaccination campaigns are cost-effective across diverse settings, with targeted strategies for vulnerable populations yielding the greatest economic and health benefits [17,18,19,20]. Testing and screening strategies, particularly frequent, low-cost PCR or antigen testing, are cost-effective in high transmission settings. Expanded symptomatic and asymptomatic testing reduces infections and deaths, with ICERs < $100,000/QALY when the effective reproduction number (Re) is high or test costs are low [21,22,23]. PCR is most cost-effective at low prevalence, while serology may be preferable at higher prevalence [22]. Routine school-based antigen testing with PCR confirmation is cost-effective during surges [23]. Social distancing and non-pharmaceutical interventions (NPIs) are cost-effective when implemented early and in combination with other measures, but their cost-effectiveness declines with prolonged duration or high economic disruption. The economic burden of strict NPIs is substantial, and their value depends on epidemic phase, compliance, and integration with vaccination and testing [6,7,23,24,25,26]. Combined strategies—rapid vaccination rollout with moderate social distancing—minimize both health and economic costs [26,27]. Vaccination—especially in high-risk groups, frequent low-cost testing, and contextually appropriate social distancing are the most cost-effective interventions for COVID-19, with optimal strategies tailored to epidemic dynamics and population risk [16,17,18,21,22,23,25,26].

The latest head-to-head economic analyses indicate that vaccination is the most cost-effective intervention for COVID-19 across healthcare settings, with testing and social distancing strategies showing variable cost-effectiveness depending on context and epidemic phase. In hospitals, improvements in therapeutics and care (hospital-based treatment and care improvements, Hospital-based treatment and care improvements (HTCI)) are highly cost-effective, with cost per QALY gained far below standard thresholds. Vaccination is also cost-effective, while non-medical interventions (non-medical interventions (NMIs), e.g., broad social distancing) are less cost-effective due to high economic and societal costs. Combining hospital-based care improvements with vaccination provides additional benefit, but adding broad NMIs increases costs substantially without proportional QALY gains [24]. In long-term care facilities (nursing homes), routine antigen testing of staff is not cost-effective under low-severity (e.g., Omicron) conditions but becomes cost-effective or even cost-saving when the risk of severe outcomes is high. The cost-effectiveness of testing is highly sensitive to the prevailing variant’s virulence and the background level of vaccination and masking. Thus, resource allocation should be dynamically adjusted based on circulating variants and resident vulnerability [28]. In schools, broad social distancing and closures are effective but among the least cost-effective interventions due to high indirect costs (e.g., lost educational attainment and future earnings). Targeted testing and vaccination strategies are more cost-effective, especially when implemented early and in combination. Community screening and surveillance in schools are cost-effective, particularly during high transmission periods, but the economic burden of prolonged closures is substantial [6,25,29].

Across all settings, the optimal strategy is a combination of rapid vaccination rollout, targeted testing, and context-appropriate social distancing, tailored to the risk profile and epidemic phase. The cost-effectiveness of each intervention is highly context-dependent, and dynamic adjustment is necessary as viral characteristics and population immunity evolve [6,18,25,27,28,29,30].

### 3.1. Cost-Effectiveness Variations Across Different Healthcare Settings

The economic value of COVID-19 interventions varies markedly between hospitals, long-term care facilities, and schools. Social distancing and school closures are effective for reducing transmission but among the least cost-effective measures because of substantial indirect costs such as lost education and future earnings. Their cost-effectiveness is highly sensitive to epidemic phase, duration, and compliance and declines as community transmission drops and depends strongly on epidemic phase and compliance levels [6,23,25,31,32]. In hospitals, broad non-medical restrictions are less efficient than medical innovations or vaccination, which deliver greater health benefits at lower cost per QALY [24,33]. In long-term care, the value of distancing and staff testing depends on variant severity and resident vulnerability—routine testing is not cost-effective during mild outbreaks (e.g., Omicron) but becomes cost-saving under high-severity conditions [28,34]. Overall, the cost-effectiveness of non-pharmaceutical interventions is highly context-dependent, reflecting differences in indirect costs, population risk, and epidemic dynamics [6,23,24,25,28,29,31,32,33,34]. Across all settings, mRNA vaccines remain the most cost-effective preventive strategy, particularly for older adults and high-risk groups. Viral vector vaccines are generally less favorable but may still reduce mortality in select populations. Vaccine cost-effectiveness is lowest in schools due to the low risk of severe disease. All vaccines demonstrate strong safety profiles, supporting the prioritization of mRNA platforms for resource allocation in high-risk environments (Table 3) [16,17,35,36,37,38,39,40,41,42].

Current evidence confirms that mRNA vaccines represent the benchmark for cost-effectiveness in COVID-19 prevention across hospitals, long-term care facilities, and schools. Viral vector vaccines are generally less favorable but still beneficial, particularly for reducing mortality among high-risk groups [37,38]. In hospitals and long-term care settings, mRNA vaccines (BNT162b2, mRNA-1273) consistently show higher effectiveness against infection, hospitalization, and death, and lower ICERs compared with viral vector products (Ad26.COV2.S, ChAdOx1). Annual vaccination of adults aged ≥ 65 years is often cost-saving and well below conventional willingness-to-pay thresholds, especially in high-risk populations [40,43]. In schools, the cost-effectiveness of vaccination is less favorable for both mRNA and viral vector vaccines due to the lower risk of severe outcomes in children and adolescents. ICERs for mRNA vaccines in these populations are higher and less robust, but targeted vaccination of high-risk students remains cost-effective [40,43]. Across all settings, mRNA vaccines are preferred due to superior effectiveness and safety profiles, with rare serious adverse events [38,41]. The cost-effectiveness of vaccination is most pronounced in older adults and those with comorbidities, and is sensitive to epidemic phase, vaccine price, and VE against circulating variants [17,40,43].

### 3.2. Cost-Effectiveness Implications of Different Vaccine Types and Dosing Strategies (Primary Series, Boosters) in High-Risk Versus Low-Risk Populations Within Each Setting

The cost-effectiveness of different coronavirus disease 2019 vaccine types and dosing strategies—including primary series and booster doses—varies substantially by risk group and healthcare setting. Across healthcare settings, mRNA vaccines (BNT162b2, mRNA-1273) remain the most cost-effective option for high-risk groups—older adults, individuals with comorbidities, and immunocompromised patients—particularly when applied through annual or biannual booster programs. For adults aged ≥ 65 years, annual vaccination typically yields ICERs well below conventional willingness-to-pay thresholds and is often cost-saving, especially during high-transmission periods or under waning immunity [16,40,43,44]. Consequently, the Advisory Committee on Immunization Practices recommends prioritizing these populations for primary and booster vaccination [16]. In contrast, vaccination of low-risk populations (younger adults, children, and healthy individuals) is less economically favorable. For these groups, ICERs frequently exceed accepted thresholds because of the lower probability of severe outcomes and modest incremental benefit [16,17,40,43,44]. Expanding coverage or increasing booster frequency in low-risk populations reduces overall efficiency. Booster doses remain most valuable for high-risk individuals, providing significant protection against severe disease, especially amid emerging variants with immune escape. More frequent boosting (e.g., every 6 months) can be justified in such cohorts, whereas it offers limited value in low-risk groups [34,40,44,45,46]. Vaccine platform also influences outcomes: mRNA vaccines consistently outperform viral vector vaccines in effectiveness and cost-effectiveness, particularly for preventing hospitalization and death [38,46]. All platforms show strong safety profiles with rare serious adverse events [38]. Overall, targeted mRNA vaccination and periodic boosting in high-risk populations—especially in hospitals and long-term care facilities—represent the most economically efficient strategy, while broad vaccination or frequent boosting of low-risk groups provides limited incremental benefit [16,17,38,40,43,44,45,46].

### 3.3. The Impact of Intervention Timing (Early vs. Late Implementation) on Cost-Effectiveness, Especially for Social Distancing and Testing Strategies

The timing of intervention implementation is a key determinant of both clinical and economic outcomes. Across hospitals, long-term care facilities, and schools, early adoption of social distancing and testing—before widespread community transmission—has been shown to be significantly more cost-effective than delayed action [25,29,47,48,49]. In hospitals and long-term care facilities, early initiation of physical distancing and testing reduces peak healthcare demand, prevents system overload, and maximizes health benefits per unit cost. Delayed interventions result in higher case counts, increased resource utilization, and diminished cost-effectiveness, as the opportunity to prevent exponential spread is lost [25,47,48]. In schools, prompt testing and distancing during surges minimize unnecessary closures and maximize QALYs gained per cost, while delayed measures increase disruption and lower effectiveness [23,48]. Modeling studies consistently confirm that interventions are most efficient when implemented soon after outbreak onset, with cost-effectiveness declining sharply as incidence rises [29,47,49].

### 3.4. The Phase of the Epidemic (e.g., Surge vs. Low Transmission) and the Relative Cost-Effectiveness Alteration of Routine Testing and Social Distancing in Schools and Long-Term Care Facilities

The phase of the coronavirus disease 2019 epidemic—specifically, periods of surge versus low transmission—has a major impact on the relative cost-effectiveness of routine testing and social distancing interventions in schools and long-term care facilities. During surge periods characterized by high transmission or the emergence of more virulent variants, both interventions become substantially more cost-effective. In schools, frequent antigen testing (e.g., twice weekly with PCR confirmation) reduces infections and missed school days at lower cost per QALY than infrequent or no testing [23,50,51]. Temporary remote or hybrid instruction can further reduce transmission and hospitalizations [52,53]. In long-term care, routine staff and resident testing and enhanced distancing (e.g., restricted visits, suspended group activities) are cost-effective only when the risk of severe outcomes is high [28,54,55,56]. Conversely, during low transmission phases, the value of intensive measures declines. Targeted or less frequent testing (e.g., symptomatic or surveillance-based) is more efficient, while broad distancing measures or closures yield limited benefit and higher indirect costs [23,50,51,53]. In long-term care, routine testing and strict distancing are generally unwarranted unless vaccine coverage is low or severe outcomes remain likely [28,34,54,56]. Overall, adaptive, context-specific strategies—intensifying measures during surges and scaling back when transmission is low—optimize both resource use and societal outcomes [23,28,34,50,51,52,53,54,55,56].

### 3.5. Comparative Cost-Effectiveness of Vaccine Platforms and Dosing Strategies by Population Risk and Setting

#### 3.5.1. Overview of Cost-Effectiveness in Adult Vaccination

Economic evaluations consistently show that adult vaccination against respiratory pathogens—such as influenza, pneumococcal disease, and COVID-19—is highly cost-effective, particularly in older adults and individuals with comorbidities. For COVID-19, vaccination and booster programs in high-risk groups produce ICERs well below conventional willingness-to-pay thresholds. Comparable results have been reported for influenza and pneumococcal vaccines, where age- and risk-based strategies deliver strong health and economic benefits [57,58,59,60].

#### 3.5.2. Vaccine Types and Dosing Strategies

Among COVID-19 vaccines, mRNA platforms (BNT162b2, mRNA-1273) are consistently more cost-effective than viral vector vaccines, reflecting higher effectiveness and favorable safety profiles [16,20,43,45]. For pneumococcal disease, PCV20 is the most cost-effective option for adults ≥ 65 years and for those with chronic conditions, with age-based use often being cost-saving [60]. Dosing frequency also influences value: annual or biannual vaccination in high-risk adults remains cost-effective, especially as SARS-CoV-2 becomes endemic and immunity wanes [20,43,45].

#### 3.5.3. High-Risk vs. Low-Risk Populations

The economic advantage of vaccination is concentrated in high-risk groups—adults ≥ 65 years, individuals with chronic conditions, and immunocompromised patients—where ICERs can be as low as $23,308 per QALY for COVID-19 and annual influenza vaccination is often cost-saving for adults ≥ 50 years [16,43,59]. In low-risk populations (healthy younger adults and children), ICERs frequently exceed $100,000 per QALY, showing high sensitivity to assumptions about disease incidence and vaccine pricing [16,43]. Expanding boosters to these groups yields limited incremental benefit and lower economic efficiency [43,45].

#### 3.5.4. Healthcare Setting Considerations

Cost-effectiveness differs across settings. In hospitals and long-term care facilities, targeted vaccination and boosting of high-risk cohorts provide the best value, substantially reducing morbidity and mortality [20,45]. In community programs, vaccination of uninsured or high-risk adults remains cost-effective even with incomplete series [61]. For pneumococcal vaccination, expanding coverage to adults aged 50–64 years is particularly favorable among subgroups with higher disease burden, such as Black adults [62].

#### 3.5.5. Sensitivity Analyses and Key Drivers

Key determinants of cost-effectiveness include vaccine price, effectiveness, disease incidence, program timing, and indirect benefits such as reduced transmission and productivity losses [20,43,58]. Sensitivity analyses consistently show robust cost-effectiveness in high-risk populations and during periods of elevated transmission. Incorporating indirect effects and real-world evidence (RWE) further strengthens the economic case for vaccination [20,43].

#### 3.5.6. Gaps and Limitations

Further country-specific and real-world data are needed on long-term booster strategies as viral evolution and population immunity change. Evidence remains limited for subpopulation-specific cost-effectiveness, and future analyses should include indirect benefits and long COVID outcomes to better capture the full societal value of vaccination [20,43]. Overall, mRNA COVID-19 vaccines and PCV20 are the most cost-effective when targeted to older and high-risk adults, with periodic boosting improving outcomes. Broader application to low-risk groups yields diminishing returns and lower economic efficiency [16,20,43,45,59,60,61,62].

#### 3.5.7. Lessons from Adult Immunization Economics

Evidence from pre-pandemic adult immunization programs provides valuable context for interpreting the economic findings of COVID-19 vaccination strategies. Across influenza, pneumococcal, and herpes zoster vaccines, cost-effectiveness was consistently enhanced when analyses adopted a societal perspective, capturing productivity gains and caregiver burden. Studies that included herd-immunity externalities or reduced hospitalization spill-overs reported substantially lower incremental cost-effectiveness ratios compared with payer-only evaluations. Furthermore, equity-oriented extensions—such as age, comorbidity, or income-stratified analyses—demonstrated that immunization programs often deliver disproportionate benefits among high-risk or underserved groups. These patterns parallel the COVID-19 context, underscoring how comprehensive perspectives, inclusion of indirect effects, and equity weighting can materially alter economic conclusions. Collectively, prior adult-vaccine experience reinforces that broad, socially informed frameworks yield more realistic estimates of value during infectious-disease crises.

### 3.6. Compliance Rates and Population Heterogeneity (Age, Comorbidities, Socioeconomic Status) Effect on the Cost-Effectiveness of Interventions

Compliance levels and population heterogeneity—including age, comorbidities, and socioeconomic status—strongly influence the cost-effectiveness of COVID-19 interventions such as vaccination, testing, and social distancing in schools and long-term care facilities. High compliance with preventive measures (masking, distancing, testing) leads to greater reductions in transmission and improved economic efficiency, whereas poor adherence diminishes both health and cost outcomes. Schools with strong health infrastructure and higher socioeconomic status generally achieve better compliance and more favorable cost-effectiveness profiles. In contrast, resource-limited or high-poverty schools face lower uptake and weaker intervention impact [63,64]. In long-term care facilities, older adults and residents with comorbidities benefit most from vaccination and testing, making these interventions especially cost-effective when the risk of severe outcomes is high [23,27,50,53]. In schools, testing and vaccination yield the greatest value in settings with low baseline vaccination, limited mitigation, or high transmission, while their benefits diminish in well-vaccinated or low-risk populations [23,50,53]. Socioeconomic disparities further shape outcomes: underserved groups—those facing low education, unemployment, or minority status—often have reduced participation in vaccination and testing, limiting both effectiveness and economic value [65]. Targeted and resource-intensive strategies that address these inequities can enhance compliance, improve health outcomes, and optimize cost-effectiveness in vulnerable populations [28,63,64,65].

### 3.7. The Role of Combined Interventions (e.g., Vaccination Plus Targeted Testing and Partial Social Distancing) Compared to Single Strategies in Maximizing Cost-Effectiveness

Modeling and cost-effectiveness analyses consistently demonstrate that combined interventions, such as vaccination plus targeted testing and partial social distancing, are more effective and cost-efficient than single strategies for COVID-19 prevention in schools and long-term care facilities [29,34,66,67,68,69,70]. In schools, agent-based models and economic analyses show that layering vaccination with regular screening (e.g., weekly PCR or antigen testing of unvaccinated students) and partial distancing protocols (such as “test-to-stay” policies) substantially reduces cases and minimizes school closures compared to symptom-based testing or distancing alone [23,50,67]. These combined strategies are especially effective during surges of highly transmissible variants, where weekly testing of 75% of unvaccinated students, in addition to vaccination, can reduce cases by up to 34–36% and student-days lost by up to 80% compared to reactive class closures [67]. The cost per infection averted and per QALY gained is lowest when these interventions are implemented together, particularly in settings with lower baseline vaccination or mitigation adherence. “Test-to-stay” policies, which allow exposed students to remain in school with frequent testing, maintain in-person learning at a lower societal cost than remote or hybrid models, and are most efficient when combined with vaccination and other mitigation measures [51,67]. In long-term care facilities, vaccination of residents and staff is the single most impactful measure for reducing hospitalizations and deaths, but routine staff testing and partial distancing (e.g., cohorting, entry regulation) further decrease outbreaks and transmission when layered with vaccination [34,68]. Multifaceted approaches—combining vaccination, regular testing, cohorting, and entry regulation—are consistently more effective and cost-efficient than any single measure, especially in high-risk populations and during periods of elevated community transmission [34,68]. Weekly routine testing of staff, when added to baseline measures, reduces resident infections by at least 25%, but vaccination averts 2–4 times more infections and nearly all hospitalizations and deaths; the greatest impact is achieved when both are implemented together [68].

The synergy between interventions enables flexible adaptation to epidemic phase, risk profile, and available resources. Modeling studies show that different combinations can achieve similar reductions in hospitalizations and transmission, allowing policymakers to tailor responses efficiently [66,69,70]. Importantly, early implementation of combined strategies maximizes cost-effectiveness by preventing exponential spread and reducing both health and economic burdens [49,70]. Despite robust modeling support, real-world data—especially from U.S. long-term care settings—remain limited. Future research should define optimal combinations and implementation pathways across diverse populations, accounting for compliance, resources, and evolving viral dynamics. Overall, combined interventions outperform single measures in reducing transmission, hospitalizations, and societal costs, particularly during surges and in high-risk groups [29,34,50,66,67,68,69,70].

### 3.8. The Impact of Non-Health Impacts and Distributional Effects (e.g., Equity, Access Disparities) in Modifying the Assessment of Cost-Effectiveness for These Interventions

Non-health impacts and distributional effects—such as equity, access disparities, and social consequences—significantly influence the cost-effectiveness of combined interventions like vaccination, targeted testing, and partial social distancing in schools and long-term care facilities. When these broader outcomes are incorporated, the perceived value of interventions often increases, as they help sustain educational continuity, prevent productivity losses, and minimize social disruption. These benefits are particularly important where school closures or strict distancing exacerbate inequalities, disproportionately affecting disadvantaged populations. Including such factors in economic evaluations, as recommended by the Second Panel on Cost-Effectiveness in Health and Medicine, yields a more comprehensive societal perspective [71,72]. Equity-sensitive analyses indicate that combined interventions provide greater health and financial protection for groups with higher disease burden and lower baseline access—such as low-income, minority, or rural populations [73,74]. Targeted vaccination and testing in underserved schools or care facilities can avert more deaths and prevent catastrophic health expenditures, enhancing both efficiency and fairness. Conversely, inadequate attention to access barriers—like vaccine hesitancy, logistical constraints, or lack of culturally tailored outreach—can inadvertently widen disparities [8,75,76]. Frameworks such as distributional cost-effectiveness analysis (DCEA) and extended cost-effectiveness analysis (ECEA) enable quantification of these equity impacts but require robust subgroup data and remain challenging to operationalize [75,76,77,78]. Neglecting non-health and equity effects risks undervaluing interventions and misdirecting resources. Incorporating these dimensions reveals the broader societal value of combined interventions and underscores the need for equity-oriented design and implementation, especially in vulnerable school and long-term care populations [8,71,72,73,74,75].

## 4. Discussion

The growing body of economic evidence on coronavirus disease 2019 (COVID-19) provides crucial insights into the efficiency, affordability, and sustainability of public health responses across diverse settings. Reviewed studies consistently show that COVID-19 has imposed an unprecedented global economic burden, with indirect costs—including productivity losses, social disruption, and long-term morbidity—often exceeding direct healthcare expenditures [1,2,3,4,5,6,7,9,10]. Conversely, well-designed interventions such as vaccination, routine low-cost testing, and targeted social distancing have proven to be cost-effective, and in some cases cost-saving, particularly when implemented early and directed toward high-risk populations [17,18,19,23,25,26,47,48]. These findings have important implications for strengthening health system resilience, improving pandemic preparedness, and guiding evidence-based resource prioritization in future global health emergencies.

From a health economics perspective, vaccination—especially with messenger RNA (mRNA) platforms—emerges as the most cost-effective intervention across healthcare settings [17,18,19,26,47]. The superior vaccine effectiveness and safety of BNT162b2 and mRNA-1273 translate into incremental cost-effectiveness ratios (ICERs) well below conventional willingness-to-pay thresholds, particularly in older adults and individuals with comorbidities [17,18,26]. Targeted booster programs for high-risk populations consistently yield substantial health gains at acceptable cost levels, whereas universal booster expansion to low-risk groups produces diminishing returns [18,19]. These findings align with core principles of health technology assessment (HTA), emphasizing the maximization of population health benefit per monetary unit spent [1,5,6]. However, vaccine cost-effectiveness remains sensitive to acquisition price, coverage rates, and evolving epidemiological conditions, underscoring the need for adaptive, data-driven vaccination strategies and real-time evaluation frameworks to optimize allocation as the pandemic evolves [17,18,19,26,47].

Economic modeling further demonstrates that timing, intensity, and combination of interventions critically determine both cost-effectiveness and societal value [3,4,6,13,17,18,25,26]. Early implementation of testing, isolation, and social distancing during epidemic surges proved substantially more efficient than delayed or reactive responses, preventing exponential transmission, hospital overcrowding, and workforce disruption [3,6,17,18]. In contrast, adaptive or de-escalated strategies—such as targeted testing or partial distancing—were more efficient during low-transmission periods, when the marginal benefits of strict measures declined [4,18,23,25]. These temporal and contextual dynamics highlight the need for flexible, evidence-based public health planning, with interventions optimized continuously according to real-time surveillance, variant characteristics, and system capacity [6,17,18,23,25,26].

At the healthcare system level, the reviewed evidence reveals substantial contextual variation in the economic performance of COVID-19 interventions [1,2,3,4,6,7,8,9,17,18,19,23,25,26]. In hospitals, vaccination and advances in clinical management—such as optimized ICU utilization, early therapeutics, and improved triage—dominate the cost-effectiveness landscape [2,3,17,18]. In long-term care facilities, vaccination of residents and staff is consistently cost-saving, while routine staff testing is economically justified primarily during high-prevalence periods or when vaccine coverage is limited [2,6,7,18,23]. In educational settings, broad school closures and prolonged distancing impose high indirect costs—through learning loss, reduced parental productivity, and mental health impacts—rendering them less favorable than targeted vaccination or testing programs [1,8,9,17,18,25,26]. Together, these findings reaffirm that cost-effectiveness is inherently context-dependent, requiring assessment frameworks that integrate both direct health outcomes and broader societal effects.

Evidence also highlights the value of combined or layered strategies. Vaccination alone, while effective against severe outcomes, cannot fully suppress transmission in partially immune populations [1,3,9,24]. Conversely, testing and distancing alone are insufficient to prevent outbreaks or maintain societal stability [6,17,29,41]. Integrated approaches—combining vaccination, regular testing, and partial distancing (e.g., “test-to-stay” in schools or entry regulation in long-term care)—consistently outperform single measures in both health and economic outcomes [20,37,45,49,56,61]. The synergy between interventions enables flexible adaptation: similar reductions in transmission and hospitalization can be achieved through different combinations, allowing decision-makers to tailor strategies to local capacities and societal tolerance for restrictions [64,67,72,76].

From a methodological standpoint, the literature demonstrates marked heterogeneity in modeling frameworks, cost inputs, and outcome measures [5,7,9,17,26]. Variation in analytic perspective (societal vs. healthcare payer), time horizon, discounting, and inclusion of indirect effects contributes to wide differences in reported results [19,20,23,29,31]. Key drivers such as productivity losses, long-term sequelae, and the economic burden of post-acute COVID-19 (long COVID) are often omitted, leading to underestimation of total burden [34,35,36,37]. Only a limited subset of studies applies distributional cost-effectiveness analysis (DCEA) or extended cost-effectiveness analysis (ECEA) frameworks, which incorporate equity and financial protection [60,61,62,63]. As a result, interventions that disproportionately benefit disadvantaged or high-risk groups may be undervalued under conventional efficiency-focused criteria [64,65,66,67]. Greater methodological standardization and transparent reporting, in line with CHEERS 2022 and the Second Panel on Cost-Effectiveness in Health and Medicine, are essential to improve comparability and policy relevance [1,68,69].

Equity and access are now recognized as integral to both efficiency and fairness in pandemic response [3,5,7,60]. Variations in compliance, population heterogeneity, and socioeconomic context influence epidemiologic and economic outcomes alike [4,9,18,62]. High adherence enhances value by maximizing health gains per cost, whereas low uptake or logistical barriers—often concentrated in underserved or rural populations—diminish efficiency and widen inequities [23,35,63,65]. Findings from equity-informed evaluations suggest that targeted investment in high-burden populations not only improves health outcomes but also enhances aggregate cost-effectiveness by preventing catastrophic expenditures and reducing long-term inequalities [64,66,67,70,71]. Hence, equity-sensitive prioritization should be a core component of both economic evaluation and pandemic preparedness, ensuring that efficiency and fairness are jointly optimized [1,68,69,72].

The policy implications are clear. Policymakers should prioritize early, targeted, and combined interventions that maximize health and economic gains while minimizing educational and societal disruption [5,7,9,17,18,26]. Investment in preparedness infrastructure—including disease surveillance, vaccine manufacturing, and rapid deployment systems—delivers long-term economic returns and strengthens resilience against future crises [29,31,34,49,51]. Expanding evaluation frameworks to include non-health impacts—such as educational continuity, workforce stability, and mental health—provides a more holistic and equitable valuation of public health measures [35,36,53,56,64,65]. As COVID-19 transitions toward endemicity, maintaining cost-effective protection for vulnerable populations through booster vaccination, adaptive testing, and resilient health systems remains essential for sustainable pandemic management [52,60,65,67,68,69,72,74].

Overall, the synthesis of available economic evidence indicates that COVID-19 interventions achieve the greatest efficiency when implemented early, targeted to high-risk populations, and layered across complementary strategies [5,7,9,17,18,23,26,47,48]. Ensuring methodological rigor, equity integration, and adaptability to changing epidemiological and socioeconomic conditions is key to sustaining both economic performance and societal value [29,31,35,60,62,64,66,69,72]. Together, these insights reaffirm that evidence-informed, equity-sensitive, and data-driven interventions are the foundation of sustainable preparedness and resilient public health systems for future global emergencies [1,34,51,63,67,74,78].

### Limitations

This review has several limitations that should be considered when interpreting its findings. First, the narrative design inherently carries a higher risk of selection and publication bias, as the search, screening, and synthesis processes are not as structured or reproducible as those of a systematic review. Second, the included studies display significant methodological heterogeneity, particularly in economic perspective, time horizon, discounting, and outcome metrics. These variations limit comparability across analyses and may contribute to inconsistencies in reported cost-effectiveness results.

Third, most economic evaluations were conducted in high-income countries, especially the United States and Western Europe, resulting in limited generalizability to low- and middle-income settings where healthcare costs, service capacity, and population characteristics differ markedly. Fourth, relatively few studies incorporated indirect or long-term consequences such as productivity loss, post-acute sequelae including “long COVID,” mental health effects, and educational disruption. Excluding these elements likely underestimates both the full economic burden of the pandemic and the value of interventions that mitigate broader societal impacts.

Fifth, equity-informative economic evaluation approaches—such as distributional or extended cost-effectiveness analyses—remain underused due to scarce disaggregated data and methodological complexity. As a result, the impact of interventions on underserved or high-risk groups may be incompletely represented. Finally, given the rapidly evolving nature of the pandemic, model-based assumptions regarding variant transmissibility, immunity duration, and behavioral adaptation may not reflect current or future epidemiological realities. Continuous evidence updates are therefore necessary to ensure long-term policy relevance.

## 5. Conclusions

This narrative review synthesizes a substantial body of global evidence demonstrating that early, targeted, and layered COVID-19 interventions—particularly vaccination, testing, and selective social distancing—offer the highest health and economic value across healthcare, community, and educational settings. Vaccination, especially with mRNA platforms, consistently emerges as the most cost-effective and often cost-saving intervention, while testing and social distancing provide complementary benefits when tailored to epidemic dynamics and population risk profiles.

The reviewed studies highlight that cost-effectiveness is context-dependent and shaped by timing, compliance, and population characteristics. Interventions implemented early in epidemic surges yield greater efficiency by averting exponential transmission and reducing pressure on healthcare systems. Conversely, broad and prolonged restrictions are less economically favorable due to substantial indirect costs. Methodological heterogeneity remains a limitation across studies, emphasizing the need for standardized analytic frameworks, transparent reporting, and inclusion of non-health impacts such as educational continuity and productivity.

Equity considerations are central to both efficiency and fairness. Economic evaluations incorporating distributional cost-effectiveness or extended cost-effectiveness frameworks demonstrate that prioritizing high-burden and underserved populations enhances overall societal value and promotes more just health outcomes. Future research should integrate real-world data, long-term consequences such as “long COVID,” and broader societal outcomes to fully capture intervention value.

Sustained investment in health system resilience, surveillance, and vaccine infrastructure will ensure preparedness for future pandemics. Integrating methodological rigor with equity-sensitive and adaptive policy design represents the cornerstone of sustainable and economically efficient public health response.

## Figures and Tables

**Table 1:** Common Methodological Weaknesses of Included Economic Evaluations.

Domain (Assessed Criterion)	Typical Shortcoming Observed	n (%) of 74 Studies
Study perspective	Perspective not explicitly stated or inconsistent with stated objectives	18 (24.3%)
Time horizon & discounting	Short horizons (<1 year) without justification; discount rates omitted	22 (29.7%)
Model structure & transparency	Incomplete reporting of model assumptions or validation	28 (37.8%)
Cost data sources	Use of aggregated or non-country-specific unit costs	31 (41.9%)
Outcome measurement	Utilities derived from secondary sources without sensitivity testing	26 (35.1%)
Uncertainty analysis	Absence of probabilistic sensitivity analysis	33 (44.6%)
Equity or distributional analysis	No stratified or equity-adjusted analyses	58 (78.4%)
CHEERS 2022 compliance	Partial adherence (<70% of items reported)	46 (62.2%)

**Table 2:** Methodological characteristics of the included economic evaluations (n = 74).

Characteristic Categories/Definitions	n (%)
Study design	
Modelling analyses (decision-analytic, compartmental, microsimulation)	68 (91.9%)
Trial-based economic evaluations	6 (8.1%)
Geographical setting	
USA/Canada	46 (62.2%)
EU/UK	21 (28.4%)
Other high-income countries	5 (6.8%)
Low- and middle-income countries (LMICs)	2 (2.7%)
Intervention types	
Vaccination interventions	41 (55.4%)
Testing and screening strategies	26 (35.1%)
Non-pharmaceutical interventions (NPIs)	21 (28.4%)
Combined/multicomponent strategies	32 (43.2%)
Economic perspective	
Healthcare payer/provider	34 (45.9%)
Societal	29 (39.2%)
Not reported	11 (14.9%)
Outcome measures	
ICER per QALY gained	50 (67.6%)
Net monetary benefit (NMB)	16 (21.6%)
Cases/hospitalizations/deaths averted	24 (32.4%)
Utility or life-years gained without ICER reporting	8 (10.8%)
Equity considerations	
Subgroup/stratified analyses	12 (16.2%)
Use of DCEA/ECEA frameworks	4 (5.4%)
Inclusion of long-term societal outcomes	
“Long COVID,” productivity losses, mental health, educational disruption	9 (12.2%)
Reporting quality	
Explicit alignment with CHEERS 2022 checklist	28 (37.8%)

**Table 3:** Summarized results about cost-effectiveness (ICER/QALY, Cost-Saving, Number needed to vaccinate (NNV)), effectiveness (VE, Hospitalization/Death Reduction) and key findings by healthcare settings.

Vaccine Type	Healthcare Setting	Cost-Effectiveness (ICER/QALY, Cost-Saving, NNV)	Effectiveness (VE, Hospitalization/ Death Reduction)	Key Findings	References
mRNA (BNT162b2, mRNA-1273)	Hospitals	ICER as low as $23,308/QALY in ≥65 years; NNV for hospitalization: 3130 (mRNA-1273), 15,472 (BNT162b2); cost-saving in high-risk	VE > 90% for symptomatic infection; highest reduction in hospitalization and death	Highest cost-effectiveness and VE, especially in older adults and high-risk; mRNA-1273 slightly superior to BNT162b2; cost-saving in some analyses	[16,17,35,36,37,38,39,40,41]
mRNA (BNT162b2, mRNA-1273)	Long-Term Care Facilities	ICER most favorable in ≥65 years and high-risk; cost-saving in targeted strategies	VE > 90% for severe disease; strong reduction in hospitalization/death	Targeted vaccination in vulnerable populations most cost-effective; mRNA vaccines preferred	[16,37,38,40,41]
mRNA (BNT162b2, mRNA-1273)	Schools	ICER > $200,000/QALY in children/adolescents; less favorable, highly sensitive to assumptions	VE high for infection, but lower for severe outcomes in youth	Effective, but cost-effectiveness less robust due to low risk of severe disease; benefit greatest in high-risk students	[16,38,40,41]
Viral Vector (Ad26.COV2.S, ChAdOx1)	Hospitals	ICER higher than mRNA; NNV for hospitalization: 26, CE.COV2.S; less cost-effective overall	VE 67–70% for symptomatic infection; strong reduction in mortality	Lower VE and cost-effectiveness than mRNA; may be more cost-effective for mortality reduction in select high-risk	[17,35,37,38,39,40,41,42,43,44,45,46,47,48,49,50,51,52,53,54,55,56,57,58,59]
Viral Vector (Ad26.COV2.S, ChAdOx1)	Long-Term Care Facilities	ICER higher than mRNA; cost-effectiveness varies by variant and risk	VE 67–70% for severe disease; mortality reduction	Less cost-effective than mRNA; may be considered in resource-limited settings or for mortality reduction	[17,37,38,41,42]
Viral Vector (Ad26.COV2.S, ChAdOx1)	Schools	ICER not well-defined; cost-effectiveness less favorable due to low severe disease risk	VE moderate for infection; low for severe outcomes in youth	Effective, but less cost-effective than mRNA; limited data for direct comparison in schools	[17,38,41,42]

## Data Availability

No new data were created or analyzed in this study.

## Abbreviations

The following abbreviations are used in this manuscript:

ACIP	Advisory Committee on Immunization Practices
CEAs	Cost-Effectiveness Analyses
CHEERS	Consolidated Health Economic Evaluation Reporting Standards
COVID-19	Coronavirus Disease 2019
DCEA	Distributional Cost-Effectiveness Analysis
ECEA	Extended Cost-Effectiveness Analysis
ICU	Intensive Care Unit
ICER	Incremental Cost-Effectiveness Ratio
HTA	Health Technology Assessment
HTCI	Hospital-based Treatment and Care Improvements
LMICs	Low- and Middle-Income Countries
mRNA	Messenger RNA
NMB	Net Monetary Benefit
NMIs	Non-Medical Interventions
NPIs	Non-Pharmaceutical Interventions
NNV	Number Needed to Vaccinate
PCR	Polymerase Chain Reaction
QALY	Quality-Adjusted Life Year
RWE	Real-World Evidence
VE	Vaccine Effectiveness

## Appendix A. Database Search Strategies

**Table A1:** Structured search strategies and outputs for PubMed, Scopus, and Web of Science.

Database	Search String (Simplified)	Limits Applied	Records Retrieved
**PubMed** (MEDLINE)	((COVID-19 OR SARS-CoV-2 OR coronavirus) AND (“cost-effectiveness” OR “economic evaluation” OR “budget impact”) AND (vaccination OR testing OR “non-pharmaceutical interventions” OR “social distancing”))	English language; humans; journal articles; 2020–2025	2323
**Scopus** (Elsevier)	TITLE-ABS-KEY((COVID-19 OR SARS-CoV-2 OR coronavirus) AND (“cost-effectiveness” OR “economic evaluation” OR “budget impact”) AND (vaccination OR testing OR “non-pharmaceutical interventions” OR “social distancing”)) AND PUBYEAR > 2019 AND PUBYEAR < 2026 AND (LIMIT-TO(LANGUAGE,”English”))	Article/Review; English; 2020–2025	2932
**Web of Science** (Core Collection)	TS = ((COVID-19 OR SARS-CoV-2 OR coronavirus) AND (“cost-effectiveness” OR “economic evaluation” OR “budget impact”) AND (vaccination OR testing OR “non-pharmaceutical interventions” OR “social distancing”))) Refined by: DOCUMENT TYPES (ARTICLE OR REVIEW) AND LANGUAGES (ENGLISH) Timespan: 2020–2025 Indexes: SCI-EXPANDED, SSCI, A&HCI, ESCI	Article/Review; English; 2020–2025	1266
